# Treatment instability in generalized anxiety disorder (GAD): a U.S. real-world evidence study

**DOI:** 10.1017/S1092852926101059

**Published:** 2026-06-17

**Authors:** Derek Louie, Erin Ferries, Susan Suponcic, Katie Robinson, Felix Lam, Abigail Silber, Matthew O’Hara, Phong Duong, Daniel Karlin, Jeffrey R. Strawn, Roger S. McIntyre

**Affiliations:** 1 Definium Therapeutics Inc, USA; 2 Value and Access Advisors, LLC., USA; 3 Trinity Life Sciences, USA; 4 Department of Psychiatry, Tufts University School of Medicine, USA; 5 University of Cincinnati College of Medicine Department of Psychiatry and Behavi, USA; 6 Department of Psychiatry, University of Toronto, Canada

**Keywords:** Anxiety, GAD, antidepressant, SRI, benzodiazepine

## Abstract

**Objective:**

To characterize the treatment burden of generalized anxiety disorder (GAD) using real-world data on pharmacotherapy use, treatment changes, and treatment progression.

**Methods:**

This retrospective analysis used closed claims from the Komodo Healthcare Map (2021–2024) to identify U.S. adults (≥18 years) with newly diagnosed or established GAD. Treatment patterns were assessed at the drug level.

**Results:**

Among the 259 158 newly diagnosed and 1 018 288 established GAD patients, GAD-related pharmacotherapy use during the 12 months post-index was 76% and 98%, respectively. Treatment patterns were further analyzed in 59 275 newly diagnosed and 86 920 established GAD patients with ≥1 pharmacy claim. Among newly diagnosed patients, 55% discontinued and 28% added or switched treatments; among established patients, 16% discontinued and 55% added or switched treatments. The median time to first treatment event varied by cohort and event type: discontinuation occurred at a median of 84 days in newly diagnosed and 119 days in established patients, combination therapy at 49 and 32 days, and switching at 31 and 42 days, respectively. Of those who discontinued, 57% of newly diagnosed and 26% of established GAD patients did not resume treatment; among those who restarted, the median treatment gap was 146 and 96 days, respectively. Overall, 75% of newly diagnosed and 94% of established GAD patients who underwent treatment modification experienced at least one additional therapy change during the study.

**Conclusion:**

This study underscores the significant unmet needs in the current treatment of GAD, evidenced by high rates of pharmacotherapy switching, discontinuation, and prolonged gaps in care.

## Introduction

Generalized anxiety disorder (GAD) is an early-onset, chronic, debilitating condition characterized by excessive and pervasive worry leading to clinically significant distress and impairment.^1^^,^^2^ The disorder affects a substantial and growing portion of the population, with a 3-year prevalence of 10.3% among U.S. adults,^3^ and carries profound clinical, economic, and humanistic disease burden. This stems from diminished health-related quality of life (HRQoL) and impacts on activities of daily living (ADLs), work productivity losses (WPLs), increased suicidal ideation, and greater healthcare resource utilization (HCRU) and costs.^4^^–^^8^ In addition, GAD onset commonly occurs in adolescence and early adulthood, which contributes to the long-term burden of the disease.^2^ Individuals with GAD also have an increased risk for developing additional psychiatric conditions, such as mood disorders and substance use disorders, consistent with the heterotypic continuity of GAD.^9^ GAD frequently coexists with psychiatric and non-psychiatric comorbidities, which can result in further decrements in HRQoL.^4^^,^^5^^,^^10^

Despite the substantial burden of GAD, effective treatment remains an unmet need for a significant proportion of patients.^11^ Treatment guidance is similarly fragmented. The absence of consensus-based algorithms introduces uncertainty with regard to when to initiate and how to sequence treatments. As of 2026, there are no formal, comprehensive clinical practice guidelines for GAD in the United States. Although the American Academy of Family Physicians previously developed a practice consensus for GAD and panic disorder, its recommendations for GAD management are limited.^12^ Initial treatment of newly diagnosed GAD should include psychotherapy and/or select pharmacotherapeutic agents.^12^^,^^13^ Antidepressants, particularly serotonin reuptake inhibitors (SRIs), are considered first-line pharmacotherapy for GAD.^12^^,^^13^ Additional options include benzodiazepines, atypical antipsychotics, and other antidepressants or anxiolytics, with each treatment posing specific limitations, including dependence and withdrawal concerns.^12^^,^^13^ GAD therapies often have a delayed time to maximum efficacy,^14^ and the trajectory of treatment response can vary by medication type and patient characteristics.^15^ A recent meta-analysis evaluating the use of SRIs and benzodiazepines in GAD found that the maximum benzodiazepine effect emerged more quickly than SRI-related improvement, though responses converged by 6–8 weeks.^15^

In the current treatment paradigm, there can be considerable uncertainty about whether, and for whom, GAD treatment will be effective. Current pharmacotherapies are reported to yield only modest response rates in GAD, with estimates across the literature noting that half of patients do not respond to first-line treatment, and on average, only one-third of patients achieve remission with SRIs, as defined by the absence of residual GAD symptoms and no ongoing functional impairment.^16^^–^^19^ Importantly, even over a prolonged treatment, individuals with GAD have only a modest likelihood of achieving full remission.^20^ In the longitudinal Harvard/Brown Anxiety Research Program (HARP) study, only 46% of women and 56% of men with GAD experienced remission at 8 years.^20^ These data underscore the need for mechanistically novel therapies for GAD with greater efficacy and acceptability to persons living with the disorder.

Side effects and adverse effects associated with current treatments are also common and include nausea, diarrhea, constipation, weight gain, dizziness, headaches, somnolence, and sexual dysfunction, among others.^21^^,^^22^ These effects frequently lead to discontinuation, particularly at higher doses.^21^^,^^23^ Real-world studies suggest that approximately half of patients with GAD discontinue pharmacotherapy prematurely, often within 3–4 months of initiation.^24^^,^^25^ Medication-related side effects and lack of efficacy are among the most common reasons for antidepressant discontinuation in the first months of use.^26^^,^^27^ This is a notable concern as discontinuation of antidepressants has been shown to be associated with an increased risk for relapse and discontinuation-emergent signs and symptoms in patients with anxiety and depressive disorders.^28^^–^^30^ Moreover, untreated or inadequately treated GAD may be associated with worsening severity, greater functional impairment, and increased risk of developing psychiatric and non-psychiatric comorbidities.^6^^,^^31^ Even with these persistent limitations, no new pharmacotherapies for GAD have been approved since 2007.

Collectively, GAD remains difficult to manage effectively, and the longitudinal treatment burden of GAD nonetheless remains incompletely characterized. Real-world data describing pharmacotherapy use, treatment changes, and progression across the disease course are sparse, limiting a comprehensive understanding of the patient journey and the acceptability and durability of available treatments. Accordingly, this study examined real-world treatment patterns in adults with GAD in a U.S. claims database, including pharmacotherapy use, discontinuation, switching, and treatment progression. By evaluating both newly diagnosed and established GAD, this analysis sought to characterize how pharmacologic management evolves over time and to provide additional insight into the effectiveness and tolerability of current treatment strategies beyond data derived primarily from randomized controlled trials.^32^

## Methods

### Data sources

This retrospective study used closed claims data from the Komodo Healthcare Map™, a longitudinal, de-identified, U.S. pharmacy, and medical claims database. The Komodo Healthcare Map™ dataset is comprised of approximately 120 million patients with closed, adjudicated payer complete medical and/or pharmacy claims from over 150 health plans, making it generally representative of the insured U.S. population.

### Study population

**Figure 1.**
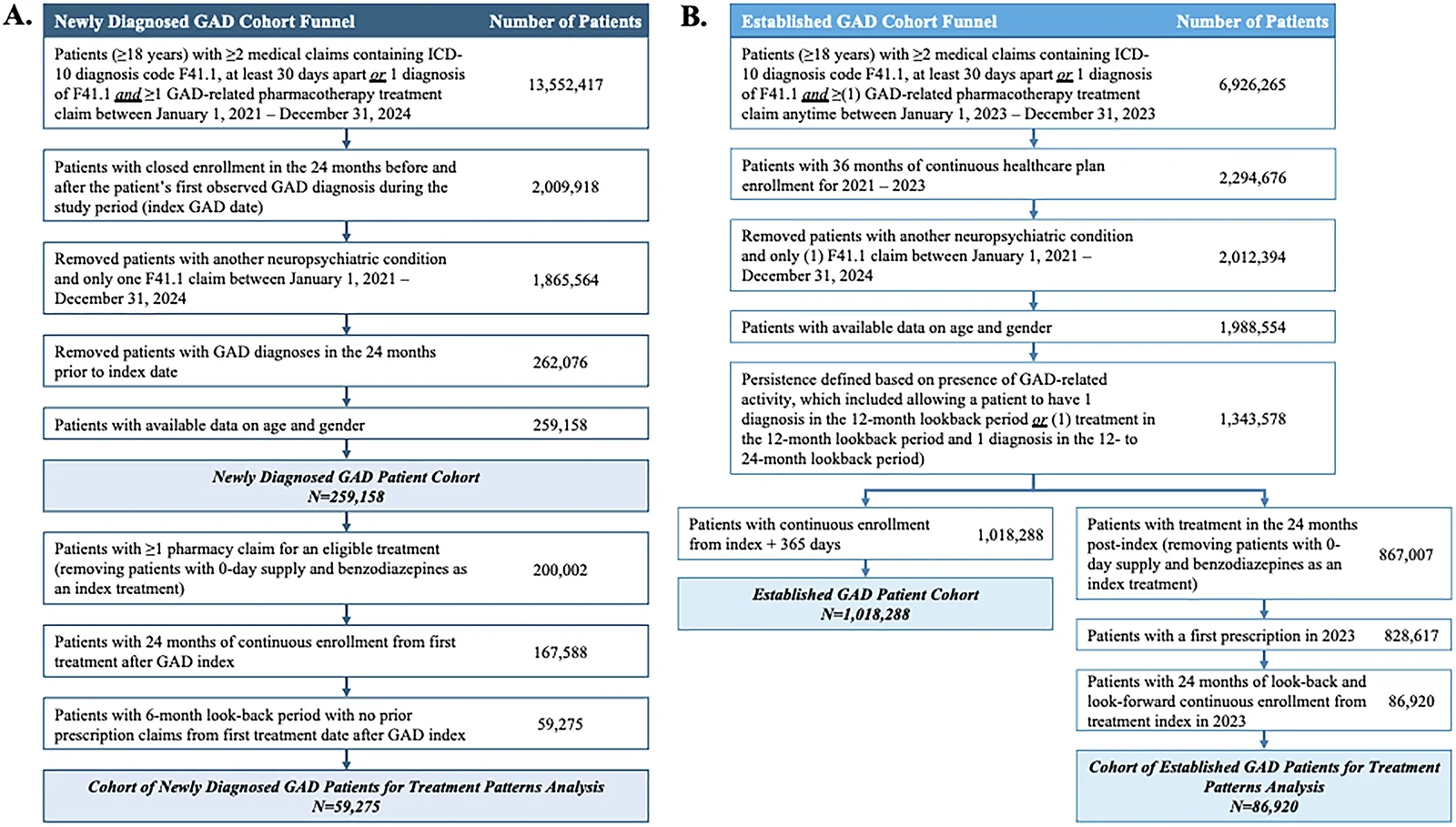
Cohort attrition. Flow diagrams illustrating selection criteria and patient attrition for the (A) newly diagnosed GAD cohort (*n* = 259 158) and (B) established GAD cohort (*n* = 1 018 288) and subset of the newly diagnosed patients (*n* = 59 275) and established GAD patients (*n* = 86 920) which were included in the treatment patterns analysis. See Supplementary Table 1 for a list of included GAD-related treatments at the drug level. Abbreviations: ICD-10, International Classification of Diseases, Tenth Revisions. Figure 1. long description.

Patients with GAD were identified using the International Classification of Diseases, Tenth Revisions (ICD-10) diagnosis code for GAD (F41.1). All patients were required to have ≥2 medical claims at least 30 days apart for F41.1 or 1 medical claim containing F41.1 and ≥ 1 GAD-related pharmacotherapy treatment claim between January 1, 2021, and December 31, 2024 (Supplementary Table 1 lists included GAD-related treatments). Patients aged <18 years at the time of the first GAD diagnosis or those missing age or gender data were excluded. Additionally, patients were excluded if they had another psychiatric condition and only (1) F41.1 diagnosis claim within the study period. Two primary GAD cohorts were defined for this study (Figure 1): a newly diagnosed GAD cohort and an established GAD cohort. In addition to the criteria noted above, patients in the newly diagnosed and established GAD cohorts were required to meet the following:

1. *Newly Diagnosed Cohort*: Index date was defined as first observed GAD diagnosis occurring between January 1, 2021, and December 31, 2024; continuous enrollment for 24 months pre- and post-index, and no GAD diagnoses in the 24 months prior to index were required.
2. *Established GAD Cohort*: Index date was defined as the first GAD diagnosis occurring between January 1, 2023, and December 31, 2023; continuous enrollment for 36 months between 2021 and 2023 and for 12 months post-index was required. This cohort was defined based on the presence of consistent GAD-related activity, including (1) F41.1 claim in the 12 months pre-index or 1 treatment claim in the 12 months pre-index and 1 F41.1 claim in 24 to 12 months pre-index.

This study was reviewed by the Advarra Institutional Review Board and was determined to have an exempt status, as defined by the U.S. Department of Health and Human Services regulations at 45 CFR 46. The authors assert that all procedures contributing to this work comply with the ethical standards of the relevant national and institutional committees on human experimentation and with the Helsinki Declaration of 1975, as revised in 2008.

### Study design

#### Patient demographics and provider characteristics

Demographic variables were collected for all newly diagnosed and established GAD patients, including age, gender, geographic distribution, and payer type (eg, commercial, Medicare, Medicaid). Healthcare provider (HCP) information was also collected for the newly diagnosed cohort to describe the providers involved in the index GAD diagnosis and subsequent management.

#### Use of pharmacotherapy and treatment patterns

Pharmacotherapy use was evaluated at the drug level for 24 months pre- and post-index by examining prescription claims for commonly prescribed GAD-related therapies (Supplementary Table 1) across the full sample of newly diagnosed and established GAD patients. For the newly diagnosed GAD patients, first-line treatment was defined as the date of the earliest GAD-related pharmacotherapy claim on or following the index date.

To characterize longitudinal disease management, GAD treatment patterns were assessed for cohorts of patients with newly diagnosed or established GAD. Inclusion in the treatment patterns analysis required meeting the following additional criteria:

1. *Newly Diagnosed Cohort*: ≥1 prescription claim for an included GAD-related treatment, continuous enrollment for 24 months following the first post-GAD-index treatment, and a 6-month look-back period with no prior prescription claims before the treatment start date.
2. *Established GAD Cohort*: Treatment during the 24 months post-index with ≥1 prescription claim for a GAD-related treatment in 2023, and 24 months of continuous enrollment before and after the treatment index in 2023.

For both cohorts, patients who received benzodiazepines as their index therapy or who had a 0-day supply of treatment were excluded from the analysis.

Treatment patterns were assessed at the drug level for 24 months following therapy initiation. Four categories were defined to capture the real-world GAD treatment pathway: discontinuation, switch, combination, and persistence (Table 1). Treatment patterns were depicted using Sankey diagrams. The diagrams illustrate the proportion of patients who remained on their initial therapy, transitioned treatments (at 0-, 90-, 180-, and 365-day post-index), or discontinued treatment. Of note, treatment changes that occurred between these predefined time points but did not extend to the subsequent time points were not captured in the Sankey diagram. Time to treatment events, lines of therapy, and therapies used during each treatment line were also assessed.

**Table 1.** Treatment Pattern Types Table 1. long description.

Treatment patterns	Description
Discontinuation	Interruption of ≥42 consecutive days of the drug regimen initiated at the treatment start date^a^
Switch	Initiation of a new drug regimen defined by a unique drug within 42 days of discontinuation of the drug regimen initiated at the index treatment date
Combination	Treatment add-on resulting in the use of ≥2 eligible GAD-related treatments simultaneously for ≥42 days
Persistence	Absence of any of the treatment changes until the end of the follow-up period^b^

a At any point, if a patient stopped treatment for ≥42 days, it was considered the end of the line of therapy. After the 42-day period, if a patient continued treatment with the same therapy, it was not considered a new line of therapy

b The patient was required to have 365 days of consecutive use from their treatment start date and have <42 days of gap between the current and next prescription (ie, script or days’ supply present in the final month)

### Statistical analysis

Descriptive statistics, including means, medians, interquartile ranges, and standard deviations, were used to tabulate results for baseline numeric characteristics and outcomes of interest. Aggregate measures of categorical variables included frequency distributions (counts and percentage of total).

### Results

#### Patient demographics and provider characteristics

A total of 259 158 patients were included in the newly diagnosed GAD cohort, and 1 018 288 patients were included in the established GAD cohort (Figure 1). Overall, 66% of newly diagnosed patients and 71% of established GAD patients were female, and 72% and 69% were commercially insured (Table 2). The mean ages of the newly diagnosed and established GAD cohort were 42 ± 17 years and 43 ± 16 years, respectively.

**Table 2.** Patient Characteristics Table 2. long description.

	Newly diagnosed cohort N = 259 158	Established GAD cohort N = 1 018 288
**Age group, % (*n*)**		
18–34	52% (135 184)	30% (302 933)
35–54	36% (92 533)	41% (421 164)
55–64	11% (27 667)	16% (164 483)
65–74	0 (0)	7% (74 112)
75+	1% (3 774)	5% (55 596)
Mean (SD)	42 (17)	43 (16)
Median (IQR)	40 (27)	40 (23)
**Gender, % (*n*)**		
Female	66% (171 554)	71% (725 143)
Male	34% (87 604)	29% (293 145)
**Geography, % (*n*)**		
Midwest	26% (67 096)	28% (281 036)
Northeast	28% (72 932)	30% (306 694)
South	29% (74 896)	27% (279 043)
West	15% (38 361)	13% (133 191)
**Payer type, % (*n*)**		
Commercial	72% (186 617)	69% (700 170)
Medicaid	14% (36 557)	15% (150 611)
Medicare	13% (34 842)	16% (165 423)

Abbreviations: IQR, interquartile range; SD, standard deviation.

Insights into the GAD patient journey, including diagnosing and treating HCPs, were captured for patients in the newly diagnosed GAD cohort. Diagnosing HCP information was available for 84% of the newly diagnosed cohort (*n* = 217 560). Among the providers, primary care physicians (PCPs) were the most common HCP included in the index GAD diagnosis claim (23%, *n* = 49 637), followed by nurse practitioners or physician assistants (NP/PA; 16%, *n* = 34 403) and internists (14%, *n* = 30 005). Collectively, psychiatrists/neurologists (reported as a single category within the claims data), psychologists, social workers, counselors, and marriage and family therapists accounted for an additional one-third of initial diagnostic encounters (38%, *n* = 83 586). Consistent with the diagnostic trends, NP/PAs (76%, *n* = 198 124), PCPs (66%, *n* = 171 264), and internists (61%, *n* = 157 115) were the leading HCPs managing patients in the 12 months post-index.

#### Pharmacotherapy use and treatment patterns

Treatment patterns are reported at the drug level in this publication; similar pharmacotherapy use patterns were evaluated at the drug class level as well. The results at the drug class level were similar to the findings reported at the individual drug level, and therefore, treatment patterns at the drug class level were not reported in this publication.

#### Newly diagnosed cohort

In the newly diagnosed GAD cohort, approximately three-quarters of patients received at least one of the GAD-related treatments included in the analysis in the 12 months post-index (76%, *n* = 197 302). The most commonly prescribed drugs were sertraline (17%, *n* = 43 741), escitalopram (17%, *n* = 43 073), bupropion (11%, *n* = 28 268), alprazolam (10%, *n* = 26 857), and trazodone (10%, *n* = 24 916).

**Figure 2.**
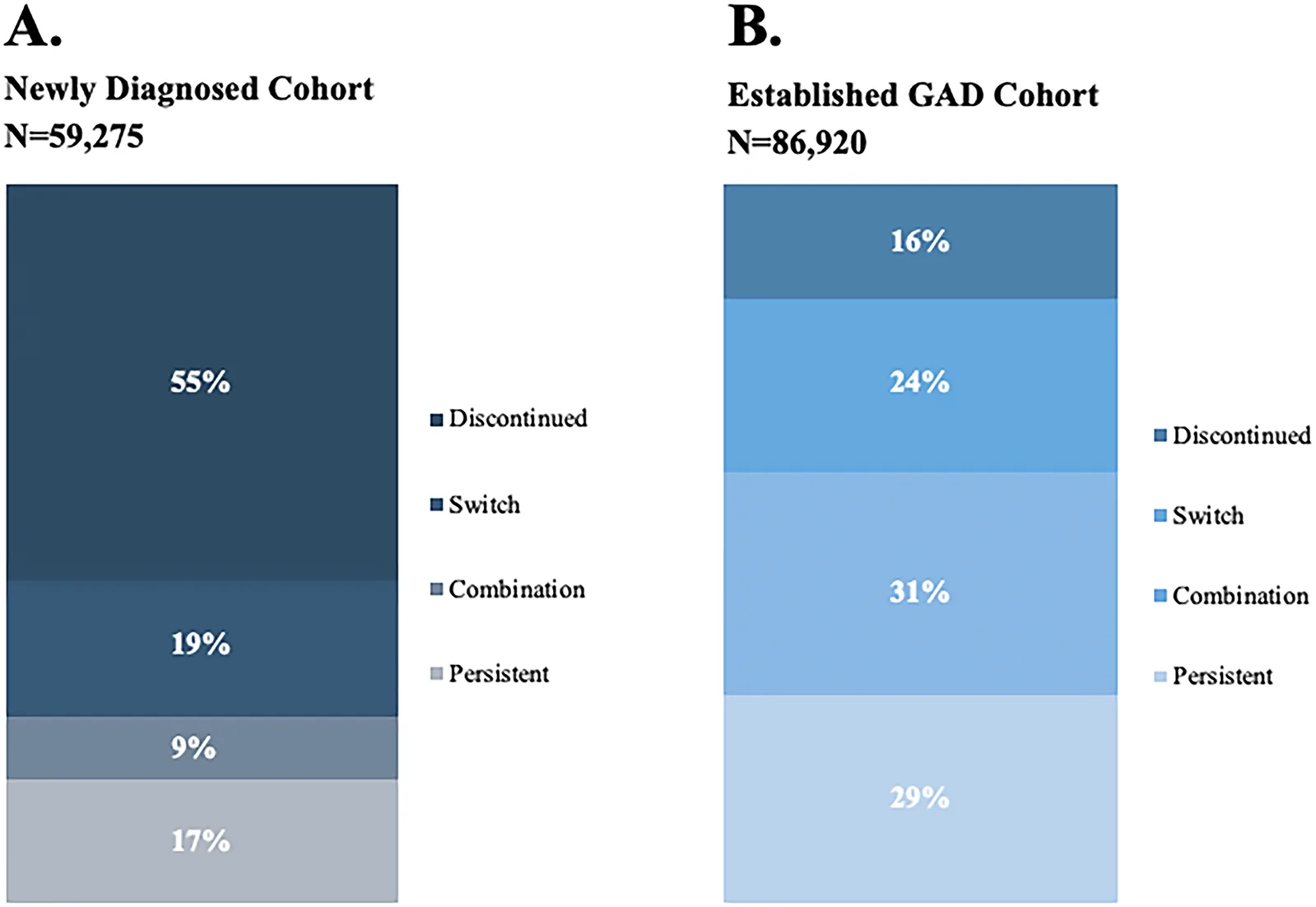
Patterns of treatment among patients with newly diagnosed and established GAD. Percent of patients in the (A) newly diagnosed GAD cohort and (B) established GAD cohort who experienced a discontinuation, switch, combination, or treatment persistence in the 12 months following their index treatment. Figure 2 long description.

Treatment progression and patterns of GAD pharmacotherapy use, including rates of discontinuation, switching, combination, and persistence, were assessed in a subset of newly diagnosed patients with ≥1 pharmacy claim for a GAD-related treatment (*n* = 59 275). Most patients (83%, *n* = 49 376) experienced a treatment change within 12 months of therapy initiation: 55% of patients discontinued treatment and 28% underwent treatment modification, defined as either switching (19%, *n* = 11 555) or combining (9%, *n* = 5 082) therapies (Figure 2). Among the patients who discontinued their first-line treatment (55%, *n* = 32 739), 43% (*n* = 13 937) resumed their use of pharmacotherapy after a median gap of 146 days (Table 3), whereas 57% ( *n* = 18 802) discontinued pharmacotherapy altogether. The median duration on initial therapy prior to discontinuation was 84 days.

**Table 3.** Time to Treatment Events Among Patients with Newly Diagnosed and Established GAD Table 3. long description.

	Newly diagnosed GAD cohort N = 59 275		Established GAD cohort N = 86 920	
	Average (SD)	Median (IQR)	Average (SD)	Median (IQR)
**Time to first event (days)**				
Discontinuation	105 (87)	84 (122)	146 (99)	119 (154)
Switching	66 (71)	31 (65)	82 (90)	42 (101)
Combination	83 (83)	49 (93)	66 (81)	32 (70)
Persistence	621 (153)	689 (275)	641 (132)	704 (197)
**Time to second event (days)**				
Discontinuation	130 (140)	90 (145)	197 (174)	129 (262)
Switching	110 (160)	38 (84)	89 (145)	29 (64)
Combination	140 (147)	87 (98)	239 (215)	147 (279)
Persistence	133 (108)	90 (128)	109 (99)	86 (134)
**Duration of first gap (days)**				
Discontinuation	209 (166)	146 (235)	150 (126)	96 (134)
Switching	11 (41)	1 (0)	8 (30)	1 (0)
Combination	10 (41)	1 (0)	4 (22)	1 (0)
Persistence	58 (65)	48 (81)	29 (48)	1 (50)

Abbreviations: IQR, interquartile range; SD, standard deviation.

**Figure 3.**
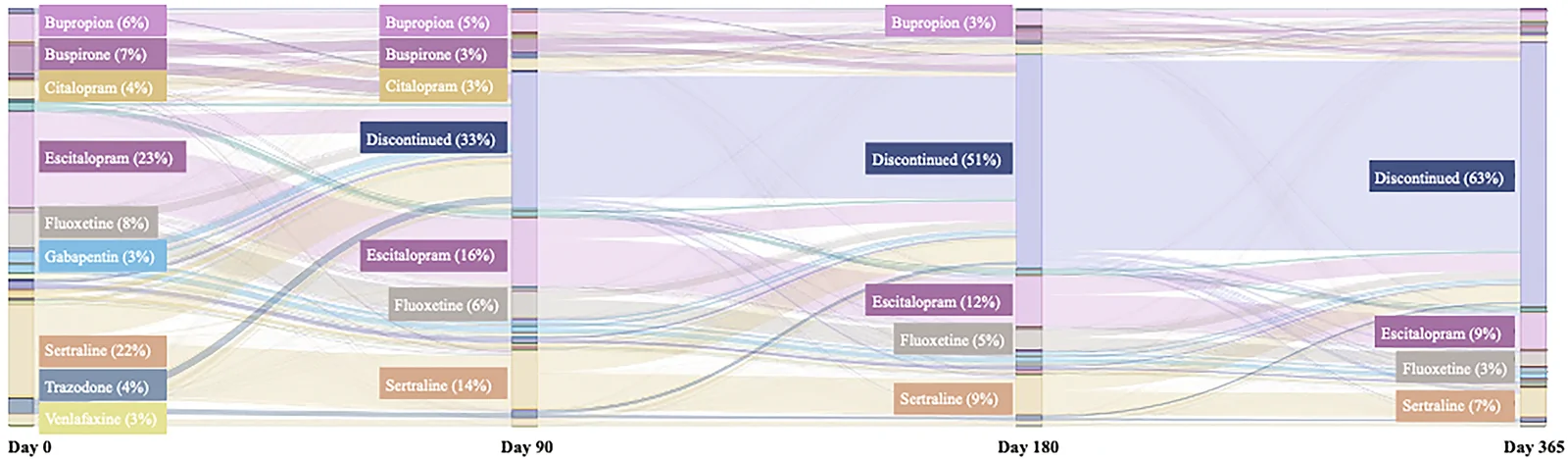
Sankey diagram of treatment patterns among patients with newly diagnosed GAD. Sankey diagram depicting the proportion of patients in the newly diagnosed GAD cohort who remained persistent with their initial therapy, transitioned between different treatments over time (at 0-, 90-, 180-, and 365-day post-index), or discontinued treatment. Each node in the diagram represents a specific treatment regimen at a defined time point, whereas the connecting flows indicate the number of patients transitioning from one regimen to another. Figure 3 long description.

For patients who switched therapy, the median time to first switch was 31 days (Table 3). More than two-thirds of patients experienced an additional treatment change a median of 38 days after starting their second therapy; subsequent changes could include an additional switch, combination, or discontinuation. Among patients who added a pharmacotherapy to their first-line treatment (9%, *n* = 5 082), the median time to combination therapy was 49 days. Nearly all of these patients experienced an additional treatment modification during the study period (94%, *n* = 4 780), which occurred at a median of 87 days after initiating their combination therapy. Overall, only 17% of newly diagnosed patients (*n* = 9 899) remained persistent with their initial pharmacotherapy for the 12 months following treatment index. As illustrated in the Sankey diagram in Figure 3, the course of pharmacotherapy in the first 12 months is frequently interrupted, predisposing patients to suboptimal treatment response.

In total, more than half of newly diagnosed patients included in the treatment patterns analysis progressed to a second-line therapy over the 24 months of follow-up (56%, *n* = 33 178). The median time to progression between the first and second treatment lines was 152 days. In addition, nearly one-third of patients (32%, *n* = 18 793) subsequently progressed to a third-line treatment.

#### Established GAD cohort

Across the established GAD cohort (*n* = 1 018 288), nearly all patients utilized pharmacotherapy in the 12 months following index (98%; *n* = 1 000 510). Established GAD patients who had ≥1 pharmacy claim for a GAD-related treatment in 2023 and who received GAD treatment in the 24 months post-index were included in the treatment patterns analysis (*n* = 86 920). As compared with the newly diagnosed GAD cohort, patients with established GAD experienced lower rates of discontinuation (16%, *n* = 13 907) but considerably higher rates of treatment modification (55%) (Figure 2). Among established GAD patients who discontinued their first-line therapy, the median duration of treatment prior to discontinuation was 119 days (Table 3). Most patients who discontinued their initial therapy subsequently restarted treatment (74%; *n* = 10 238), with a median time to restart of 96 days. However, 26% of patients (*n* = 3 533) did not resume pharmacotherapy during the study period.

The most common initial treatment change for the established GAD cohort was drug modification, including treatment switches (24%; *n* = 20 758) and combination of multiple pharmacotherapy treatments (31%; *n* = 27 220) (Figure 2). For patients who switched their initial pharmacotherapy, the median time to switch was 42 days (Table 3). Almost all of these patients experienced another treatment change during the study period (92%; *n* = 19 131) at a median of 29 days after initiating their second therapy. Among patients who added combination pharmacotherapy (31%; *n* = 27 220), the median time to combination was 32 days. Again, nearly all of these patients experienced another treatment change during the study period (96%; *n* = 26 060) at a median of 147 days. Approximately one-third of patients (29%, *n* = 25 171) remained persistent with their initial pharmacotherapy for the 12 months following treatment index. Consistent with findings for the newly diagnosed GAD cohort, there was marked variability in drug treatment patterns in patients with established GAD (Supplementary Figure 1).

Three-quarters of established GAD patients included in the treatment patterns analysis progressed to a second treatment (77%, *n* = 66 653) and two-thirds (63%, *n* = 54 595) progressed to a third treatment across the 24-month follow-up period. The median time to progression between the first and second therapies was 82 days.

## Discussion

This study provides real-world evidence characterizing pharmacotherapy and treatment trajectories throughout the GAD patient journey. There was marked instability across the treatment pathway, owing to frequent early discontinuations among patients with newly diagnosed GAD, and high rates of treatment switching among patients with established GAD. Together, these data highlight the challenges with achieving response and sustained remission with existing pharmacotherapy options.

Among those newly diagnosed with GAD, more than half discontinued treatment within 12 months of initiation. Compounding this, we found significant attrition during the acute phase of treatment (ie, initial ~12 weeks), consistent with research in other psychiatric conditions reporting that the highest rates of antidepressant discontinuation occur during this period.^33^ In the present study, most patients discontinued therapy in less than 3 months, notably short of current guidance to continue treatment for at least 6–12 months to reduce the risk of GAD relapse, representing a critical treatment gap.^12^^,^^13^^,^^30^ In the seminal trial by Rickels et al., patients with GAD who discontinued SRI treatment after only 6 months experienced significantly higher relapse rates during months 6–12 than patients who received SRI treatment for the full 12 months (53.7% vs. 9.8%; *p* < 0.001).^30^ After patients in the 12-month SRI group transitioned to placebo, relapse rates remained significantly higher for the 6-month SRI treatment group (53.7% vs. 32.4%; *p* < 0.03).^30^ Although the present study did not assess patient outcomes, the demonstrated high discontinuation rate and prolonged treatment gap suggest that patients did not achieve adequate therapeutic response with initial treatment and may have been experiencing discontinuation symptoms, further exacerbating GAD burden. Antidepressant discontinuation is associated with a risk for discontinuation-emergent signs and symptoms, such as irritability, nausea, insomnia, and dizziness, which can be frequent and severe.^29^^,^^34^^,^^35^ Moreover, since discontinuation symptoms can last ≥12 weeks and have clinical overlap with manifestations of GAD, they can be difficult to differentiate from relapse.^29^ Despite these well-established risks, treatment discontinuation is a pervasive issue in GAD, particularly among newly diagnosed patients. A previous study in the United Kingdom found a similarly high discontinuation rate of 46% and short mean treatment duration prior to discontinuation of 112 days for patients with GAD who initiated an antidepressant as their index therapy over a 9-month follow-up.^24^

Treatment discontinuation frequently resulted in prolonged interruptions in pharmacological care. More than half of newly diagnosed patients who discontinued their initial therapy did not resume pharmacologic treatment during the 24-month follow-up period. Even among those who eventually restarted therapy, the median gap between discontinuation and initiation of a subsequent treatment was nearly 5 months. While patients may have initiated or remained on non-pharmacological treatment during these gaps, these significant lapses in pharmacological care highlight the potential instability of disorder management and raise concern for sustained periods of undertreated GAD, which can lead to substantial humanistic and economic burden.^6^ In a recent analysis of the 2022 U.S. National Health and Wellness Survey, Duong et al. found that adults with likely GAD (ie, GAD symptoms, but no formal diagnosis or treatment) experienced lower HRQoL, greater WPL and ADL impairment, and higher HCRU and costs than patients with diagnosed GAD.^6^ Inadequate GAD management can also have consequences for the course and severity of comorbid conditions, which are highly prevalent in patients with GAD.^5^^,^^6^^,^^36^ A recent systematic review found that GAD adversely affects the course of multiple psychiatric and non-psychiatric comorbidities, including major depressive disorder (MDD), borderline personality disorder, social phobia, substance use, human immunodeficiency virus, and inflammatory bowel disease.^36^ Across these conditions, co-occurring GAD was associated with higher complication risks, lower remission rates, and higher HCRU and costs.^36^ These findings highlight that inadequate GAD control amplifies both clinical and economic burden beyond GAD alone.

Consistent with prior research revealing that most patients do not respond to initial GAD treatment, more than half of the newly diagnosed cohort progressed to a second-line therapy. In fact, less than one-fifth of newly diagnosed patients remained persistent with their index treatment in the 12 months after initiation. Decades of research have yet to resolve the trial-and-error nature of SRI agent selection, with each therapy offering different clinical characteristics (eg, potency/selectivity, duration of action, tolerability).^13^ In the present study, a broad range of antidepressant agents were prescribed for index treatment. While escitalopram and sertraline accounted for approximately half of first-line therapies, at least one patient in the newly diagnosed cohort received a prescription for each SRI assessed. Further complicating treatment selection, it typically takes between 6 and 8 weeks to determine if an antidepressant will be effective.^15^ Although benzodiazepines have a more rapid onset of efficacy,^15^ they are plagued by notable concerns with misuse, dependency, and withdrawal and are, therefore, recommended for only short-term, adjunctive use (~3–6 months).^12^^–^^14^ Notably, one-quarter of the newly diagnosed cohort received a non-recommended first-line treatment, including atypical antidepressants, anxiolytics, anticonvulsants, and beta-blockers. Since these therapies are typically associated with even less favorable side-effect profiles than antidepressants,^13^^,^^14^ their use may also contribute to the high discontinuation rates found in this analysis. There remains a clear need for treatments that provide better efficacy, rapid onset, sustained symptom control, and improved tolerability.

Treatment patterns in the established GAD cohort were characterized by significant instability, including frequent combinations and switches during the 12 months following index treatment. Although the precise stage of each patient’s treatment journey cannot be determined, the chronic and recurrent nature of GAD, which is characterized by modest response rates, low remission rates, and frequent symptom recurrence, suggests that the established GAD cohort likely represents a population with more difficult-to-manage anxiety.^20^^,^^37^^,^^38^ Within this cohort, pharmacologic management appeared unstable, with high rates of combination and switching. Importantly, these modifications rarely resulted in durable stabilization on pharmacotherapy. Among established GAD patients who added a medication to their index therapy, 96% underwent an additional treatment change during follow-up. Similarly, 92% of those who switched treatments required subsequent modification. Even among patients with newly diagnosed GAD, treatment changes occurred early, with index therapies often maintained for only ~4–6 weeks before switching or combination. Collectively, these findings suggest that current pharmacologic strategies, whether through combination or substitution, frequently do not provide sustained symptom control, underscoring the limitations of the existing therapeutic armamentarium for GAD.

In addition, the GAD treatment journey is often a complicated psychiatric and medical comorbidity management journey for many patients. The high and early rates of combination therapy, particularly in the established GAD cohort, where nearly one-third of patients added a second agent within approximately one month of index treatment, may reflect clinicians responding to emerging or residual depressive symptoms rather than inadequate anxiolytic coverage alone. The majority of pharmacotherapy combinations in this population were antidepressants with established use across both GAD and MDD, a pattern consistent with a comorbidity management model in which treatment decisions are shaped by symptom domains that extend beyond the anxiety spectrum. Under this interpretation, some proportion of the observed pharmacotherapy instability may represent titration or augmentation to address a more complex syndromic presentation.

Taken together, the patterns of treatment instability observed across both cohorts may not solely reflect the GAD-targeted pharmacotherapy journey, but also the clinical complexity introduced by high rates of co-occurring psychiatric conditions. The most notable is MDD, which co-occurs in an estimated 50–80% of GAD patients^39^^,^^40^ and has been shown to significantly reduce the likelihood of GAD recovery.^37^ Clinicians managing patients with GAD may be navigating a complicated and incompletely characterized comorbidity profile, where emerging or inadequately treated depressive symptoms require modifications in treatment, including switches, augmentation, and combinations. While claims data cannot confirm the degree to which modifications were driven by residual or emergent depressive symptoms, the high prevalence of MDD warrants consideration as a primary effect modifier of treatment trajectories in this population and also further drives the urgency of identifying treatments that are effective in treating residual symptoms in commonly co-occurring anxiety and depressive disorders as well as in mixed anxiety and depressive disorders.

This analysis also found a high rate of rapid treatment progression to subsequent lines of therapy, with 32% and 63% of newly diagnosed and established GAD patients receiving third-line therapy within the 24-month study period, respectively. Although there are no formal clinical practice guidelines for GAD in the United States, recommendations from the American Academy of Family Physicians^12^ and an evidence-based treatment algorithm published by Strawn et al.^13^ can guide longitudinal management. For patients with inadequate response to first-line therapy, subsequent treatment selection is often driven by pragmatic considerations, including prior response, tolerability, symptom severity, and clinician experience.^13^ In the absence of sequential strategies of strong predictors of treatment response, clinicians are frequently left navigating a trial-and-error approach. Future research must prioritize identifying clinical, biological, and treatment response predictors to individualize care, analogous to efforts such as the landmark STAR*D trials in MDD.^41^

There are potential limitations to consider with the present study. Psychotherapy represents an important component of mental health services, both as an adjunct to medications or as stand-alone treatment, and we cannot exclude the possibility that pharmacotherapy discontinuation was followed by a transition to non-pharmacological care for certain patients; however, we could not ascertain the usage reliably in the data. Recent literature suggested both low acceptance and reimbursement rates for psychotherapy and non-pharmacological care across a broad range of mental healthcare providers, and access to psychotherapy is often dependent on the patients’ self-pay, even when they have health insurance.^42^^,^^43^ An inherent limitation of claims analyses is that the data captured are restricted to information available in medical claims. The reliable tracking of prescription medication dispensing across pharmacy and medical claims, except in instances of self-pay, supports the validity of these findings as an accurate reflection of the instability in the current GAD treatment paradigm.

Additionally, this analysis did not distinguish patients with GAD alone from those with comorbid psychiatric conditions. Given that psychiatric conditions commonly co-occur among patients with GAD, this may meaningfully influence treatment trajectories. Treatment modifications that appear attributable to GAD pharmacotherapy failure may, for example, reflect clinical responses to emerging or residual depressive symptoms. Future studies linking claims data to clinical assessments of comorbid psychiatric burden would substantially advance interpretation of treatment patterns.

Our study included patients who were insured, sought care, and were continuously enrolled for at least 2 years pre- and post-index to enable longitudinal insight into the GAD treatment journey. While pharmacotherapy treatment rates may be higher in this sample than real-world surveys of GAD,^11^ the patterns of use we identified should be broadly representative of experiences with current GAD treatments. The two-year pre-index continuous enrollment period was also used to identify a population of patients with a new diagnosis of GAD. Although it is possible that some patients may have had GAD-related claims preceding this two-year window, the lookback period was retained so as not to bias the cohort toward patients with further extended, continuous insurance coverage. Additionally, while our sample contained a broad range of payer types, the under-capture of certain payers, namely, the Veterans Affairs Healthcare system, merits additional research to understand treatment patterns for this population of patients with GAD.^44^

Finally, while benzodiazepines play an important role in treatment-resistant and clinically complex GAD populations, they were excluded from the primary treatment trajectory analyses due to their distinct prescribing patterns and frequent short-term or adjunctive use. However, they remain an important component of treatment for many patients with GAD, particularly those with severe symptoms, acute distress, treatment resistance, or persistent insomnia.^45^ In real-world practice, benzodiazepines are often used intermittently or adjunctively with antidepressants, and their exclusion may, therefore, underestimate the overall complexity and pharmacologic burden associated with treatment. Future research is warranted to examine patterns of benzodiazepine use in greater depth. Importantly, our data reinforce evidence from clinical trials and real-world studies demonstrating that current treatments are inadequate and that sustained remission remains difficult to achieve with existing pharmacologic approaches.

## Conclusions

Current pharmacological management of GAD is inadequate and marked by frequent treatment modifications, high rates of discontinuation, and prolonged gaps in care among newly diagnosed GAD, and frequent treatment modifications in established GAD. The present study extends our knowledge by underscoring the significant unmet needs throughout the treatment journey, from newly diagnosed to established GAD. Given the profound burden and rising prevalence of GAD in the United States, there is an urgent need for innovative and novel pharmacotherapies that offer rapid and sustained symptom resolution.

## Supporting information

10.1017/S1092852926101059.sm001 Louie et al. supplementary material Louie et al. supplementary material

## Supplementary material

The supplementary material for this article can be found at http://doi.org/10.1017/S1092852926101059.

## Long descriptions

### Figure 1. Long description

Panel A, Newly Diagnosed G A D Cohort Funnel:

* Patients 18 years or older with 2 or more medical claims containing I C D-10 code F 41.1 at least 30 days apart or 1 diagnosis of F 41.1 and 1 or more G A D-related pharmacotherapy treatment claim between January 1, 2021 and December 31, 2024: 13,552,417.

* Patients with closed enrollment in the 24 months before and after the first observed G A D diagnosis: 2,009,918.

* Removed patients with another neuropsychiatric condition and only one F 41.1 claim: 1,865,564.

* Removed patients with G A D diagnoses in the 24 months prior to index date: 262,076.

* Patients with available data on age and gender: 259,158.

* Newly Diagnosed G A D Patient Cohort N equals 259,158.

* Patients with 1 or more pharmacy claim for an eligible treatment: 200,002.

* Patients with 24 months of continuous enrollment from first treatment: 167,588.

* Patients with 6-month look-back period with no prior prescription claims: 59,275.

* Cohort of Newly Diagnosed G A D Patients for Treatment Patterns Analysis N equals 59,275.

Panel B, Established G A D Cohort Funnel:

* Patients 18 years or older with 2 or more medical claims containing I C D-10 code F 41.1 at least 30 days apart or 1 diagnosis of F 41.1 and 1 or more G A D-related pharmacotherapy treatment claim between January 1, 2023 and December 31, 2023: 6,926,265.

* Patients with 36 months of continuous healthcare plan enrollment: 2,294,676.

* Removed patients with another neuropsychiatric condition and only one F 41.1 claim: 2,012,394.

* Patients with available data on age and gender: 1,988,554.

* Persistence defined based on presence of G A D-related activity: 1,343,578.

* This group splits into two paths.

* Path 1: Patients with continuous enrollment from index plus 365 days: 1,018,288. Established G A D Patient Cohort N equals 1,018,288.

* Path 2: Patients with treatment in the 24 months post-index: 867,007. Patients with a first prescription in 2023: 828,617. Patients with 24 months of look-back and look-forward continuous enrollment: 86,920. Cohort of Established G A D Patients for Treatment Patterns Analysis N equals 86,920.

Navigate back to Figure 1..

### Table 1. Long description

Table displays four types of potential treatment pattern assignment definitions: discontinuation, switch, combination, and persistence

Navigate back to Table 1..

### Table 2. Long description

Table that displays the patient characteristics of the newly diagnosed GAD cohort (n = 259 158) and the established GAD cohort (1 018 288)

Navigate back to Table 2..

### Figure 2. Long description

The figure consists of two panels, A and B, each representing a cohort with its sample size N and treatment outcomes categorized as Discontinued, Switch, Combination, or Persistent.

Panel A. Newly Diagnosed Cohort, N equals 59,275. The stacked bar shows the following distribution from top to bottom:

* Discontinued: 55 percent.

* Switch: 19 percent.

* Combination: 9 percent.

* Persistent: 17 percent.

Panel B. Established G A D Cohort, N equals 86,920. The stacked bar shows the following distribution from top to bottom:

* Discontinued: 16 percent.

* Switch: 24 percent.

* Combination: 31 percent.

* Persistent: 29 percent.

Navigate back to Figure 2..

### Table 3. Long description

Table displaying the time to the first treatment event, the second treatment event, and the duration of the first treatment gap

Navigate back to Table 3..

### Figure 3. Long description

The diagram tracks G A D treatment across four intervals.

At Day 0, the primary treatments are Escitalopram 23 percent, Sertraline 22 percent, Fluoxetine 8 percent, Buspirone 7 percent, Bupropion 6 percent, Citalopram 4 percent, Trazodone 4 percent, Gabapentin 3 percent, and Venlafaxine 3 percent.

At Day 90, the Discontinued category appears as the largest node at 33 percent. Remaining treatments include Escitalopram 16 percent, Sertraline 14 percent, Fluoxetine 6 percent, Bupropion 5 percent, Buspirone 3 percent, and Citalopram 3 percent.

At Day 180, the Discontinued node grows significantly to 51 percent. Active treatments continue to decline with Escitalopram at 12 percent, Sertraline at 9 percent, Fluoxetine at 5 percent, and Bupropion at 3 percent.

At Day 365, the Discontinued node reaches its peak at 63 percent. The remaining patients are on Escitalopram 9 percent, Sertraline 7 percent, and Fluoxetine 3 percent.

Flow lines indicate that while some patients switch between different S S R I or S N R I medications, the dominant flow from every treatment node at every time interval is toward the Discontinued category.

Navigate back to Figure 3..
