# Supplementary material for: Treatment instability in generalized anxiety disorder (GAD): a U.S. real-world evidence study
Source: CNS Spectr. 2026 Jun 17;31(1):e22. doi: 10.1017/S1092852926101059 (PMC13732621; doi:10.1017/S1092852926101059)
Supplement: Supplementary file 1 [file S1092852926101059sup001.docx]

**Supplementary Table 1. Eligible GAD-related Pharmacotherapies**

| **Drug Class** | **Generic Name** |
| --- | --- |
| **Selective serotonin reuptake inhibitors** | - Citalopram - Escitalopram - Fluoxetine - Paroxetine - Sertraline |
| **Serotonin-norepinephrine reuptake inhibitors** | - Desvenlafaxine - Duloxetine - Levomilnacipran - Milnacipran - Venlafaxine |
| **Atypical antidepressants** | - Bupropion - Esketamine - Mirtazapine - Nefazodone - Trazadone - Vilazodone - Vortioxetine |
| **Anxiolytics** | - Buspirone |
| **Benzodiazepines*** | - Alprazolam - Clonazepam - Diazepam - Lorazepam |
| **Monoamine oxidase inhibitors** | - Phenelzine - Tranylcypromine |
| **Antihistamines** | - Hydroxyzine |
| **Tricyclic antidepressants** | - Amitriptyline - Amoxapine - Clomipramine - Imipramine - Nortriptyline - Trimipramine |
| **Atypical antipsychotics** | - Quetiapine |
| **Anticonvulsants**** | - Gabapentin - Pregabalin |
| **Beta-blockers**** | - Atenolol - Metoprolol - Propranolol |

*Not included as an index treatment for treatment patterns analysis

**Not considered as an inclusion factor for patient identification

**Supplementary Figure 1. Sankey Diagram of Treatment Patterns Among Patients with Established GAD**

**Supplementary Figure 1.** Sankey Diagram of Treatment Patterns Among Patients with Established GAD. Sankey diagram depicting the proportion of patients in the established GAD cohort who remained persistent with their initial therapy, transitioned between different treatments over time (at 0-, 90-, 180-, and 365-days post-index), or discontinued treatment. Each node in the diagram represents a specific treatment regimen at a defined time point, whereas the connecting flows indicate the number of patients transitioning from one regimen to another.
